# Nature relatedness, flow experience, and environmental behaviors in nature-based leisure activities

**DOI:** 10.3389/fpsyg.2024.1397148

**Published:** 2024-06-06

**Authors:** Andaç Akçakese, Mehmet Demirel, Alperen Fatih Yolcu, Hüseyin Gümüş, Cihan Ayhan, Halil Sarol, Özkan Işık, Duygu Harmandar Demirel, Leonard Stoica

**Affiliations:** ^1^Department of Recreation Management, Faculty of Tourism, Necmettin Erbakan University, Konya, Türkiye; ^2^Department of Recreation Management, Institute of Social Sciences, Necmettin Erbakan University, Konya, Türkiye; ^3^Department of Recreation, Faculty of Sport Science, Mersin University, Mersin, Türkiye; ^4^Department of Recreation, Faculty of Sport Science, Sakarya University of Applied Sciences, Sakarya, Türkiye; ^5^Department of Recreation, Faculty of Sport Sciences, Gazi University, Ankara, Türkiye; ^6^Department of Coaching Education, Faculty of Sports Sciences, Balikesir University, Balikesir, Türkiye; ^7^Directorate of Sports Sciences Application and Research Center, Balikesir University, Balikesir, Türkiye; ^8^Ahmet Keleşoğlu Faculty of Education, Necmettin Erbakan University, Konya, Türkiye; ^9^Department of Individual Sports and Physical Therapy, Faculty of Physical Education and Sport, Dunarea de Jos University of Galati, Galati, Romania

**Keywords:** leisure, outdoor recreation, flow experience, nature, environment

## Abstract

**Introduction:**

Through nature-based leisure activities, spending time in nature offers opportunities to reduce stress, relax the mind, and enhance feelings of well-being. Being aware of the benefits provided by these activities increases the nature relatedness, and during the time spent in nature, it enables experiencing positive and satisfying moments by entering into a state of flow. The concepts of nature-relatedness and flow experience represent psychological experiences and characteristics that play an important role in enhancing psychological well-being and life quality.

**Methods:**

Based on structural equation models, the relationships among nature-relatedness, flow experience, and environmental behaviors were investigated. Data were collected from 379 individuals (212 male, 167 female) who regularly engage in nature-based leisure activities such as cycling, hiking, and fishing. The participants were predominantly male (55.9%) and aged 45  years and over (53.3%).

**Results:**

The nature-relatedness significantly influences flow experience (*R*^2^ = 0.505, *p* < 0.01), environmental behavior (*R*^2^ = 0.108, *p* < 0.01), environmental sensitivity (*R*^2^ = 0.137, *p* < 0.01), and communication with nature (*R*^2^ = 0.200, *p* < 0.01). Specifically, nature-relatedness directly enhanced environmental sensitivity (0.494 total effect), environmental behavior (0.604 total effect), and communication with nature (0.599 total effect) and did so both directly and indirectly through the mediation of flow experience.

**Discussion:**

A higher level of nature-relatedness can lead to a stronger flow experience, which in turn can increase positive. environmental behavior, environmental sensitivity, and communication with nature.

## Introduction

Leisure is defined as a period of time in which individuals have the opportunity to rest, have fun, and engage in personal interests and hobbies amidst the busy modern life (Veal, 2019). Leisure offers a range of benefits such as reducing stress, improving mood, encouraging creativity, and increasing overall life satisfaction (Edginton et al., 1995; Iwasaki and Schneider, 2003; Kuykendall et al., 2015; Whiting and Hannam, 2015). However, today, factors such as the rapid development of technology and the spread of urban life can reduce the impact of leisure benefits by taking people away from nature. Nonetheless, leisure is not limited to nature-based activities; enriching leisure experiences can also occur in non-natural settings such as city parks, art galleries, and museums. This demonstrates that leisure encompasses a wide spectrum, allowing individuals to explore personal interests and hobbies in both natural and man-made environments.

Nature-based activities are a natural part of leisure and offer the opportunity to interact with the natural environment. These activities include outdoor activities such as hiking, camping, mountaineering, cycling, and swimming. Nature-based activities take people away from stressful urban life and allow them to experience the peace and tranquility of nature. It also encourages physical activity, increases fitness, and supports a healthy lifestyle (Brymer et al., 2010; Lawton et al., 2017). Studies have shown that time spent in nature positively affects mental and emotional health, reduces stress, improves mood, and encourages creativity (Tinsley and Eldredge, 1995; Iwasaki et al., 2005; Li et al., 2021; Onu et al., 2022; Ratcliffe et al., 2022).

Nature possesses many elements that encourage the experience of flow. The concept of flow refers to a state of complete immersion in a physical or cognitive action (Csikszentmihalyi, 1988). According to the Attention Restoration Theory, time spent in nature helps restore the ability to apply directed attention, thus alleviating mental fatigue (Csikszentmihalyi, 1988). The “soft” fascination of nature allows directed attention to rest, enhancing an individual’s ability to focus on tasks they might not inherently find interesting or fascinating (Kaplan and Kaplan, 1989; Berman et al., 2008). This restoration process facilitates the transition into a flow state, as flow requires reaching the highest levels of concentration and motivation during activities that hold an intrinsic fascination for the individual (Csikszentmihalyi, 1988; Nakamura and Csikszentmihalyi, 2002). In this context, it can be stated that nature-based leisure activities enhance the flow experience (Nakamura and Csikszentmihalyi, 2002; Göker, 2022).

People who are in flow in activity in nature tend to exhibit behaviors that respect the natural environment, protect the environment, and fulfill their environmental responsibilities (Barbaro and Pickett, 2016; Rosa et al., 2018). The experience of flow increases intrinsic motivation and this contributes to more positive and sustainable environmental behaviors (Csikszentmihalyi and Rathunde, 1993; Faraz et al., 2021). In addition, people in the flow in nature lose their ties with the outside world and interact with their environment in a more natural way (Howell et al., 2011). Environmental sensitivity increases during activities in nature, because interaction with the beauty and sensitivity of the environment increases awareness of nature. This contributes to the development of an attitude towards protecting nature (Lee et al., 2013; Lee and Jan, 2015). In addition, when people are in the flow, they communicate more openly, understandingly, and sincerely with their environment (Kim et al., 2020). Nature has a calming and therapeutic effect on people; this effect is further strengthened by the flow experience. Thus, people better understand the natural environment and develop a greater intrinsic motivation to protect it (Capaldi et al., 2014; Xie et al., 2022).

The concepts of nature-relatedness and flow experience are important concepts for understanding people’s relationship with the natural environment and their positive psychological experiences. In particular, examining how flow experience is formed is an important and complex proposition in terms of scientific literature. This study aimed to create and validate a model that tests the relationship between nature-relatedness and the variables of environmental behavior, environmental sensitivity, and communication with nature for individuals engaged in nature-based leisure activities by using flow experience as a mediating variable. In this way, the existing theory of nature-relatedness and flow experience can be improved, contributing to the development of effective strategies for environmental education and increasing awareness of the natural environment, and supporting sustainability efforts.

## Literature review

### Nature relatedness

Nature-relatedness is a psychological construct that explains the subjective relationship between the natural environment and individuals, encompassing cognitive, emotional, and experiential aspects of individuals’ interaction with nature (Huang et al., 2022). It is characterized by feelings of gratitude, understanding, and commitment to the natural world (Sadowski et al., 2022), emphasizing the recognition of oneself as a part of nature and feeling responsible for other living things and the environment (Yurtsever and Angın, 2021). Research has shown that individuals with a higher connection to nature are more concerned about environmental issues and more likely to engage in eco-friendly behaviors, considering the impact of their actions on the environment (Wilkie, 2019; DeVille et al., 2021). Furthermore, nature-relatedness is associated with physical activity and participation in leisure activities (Molina-Cando et al., 2021), where leisure activities can increase nature-relatedness by encouraging interactions with natural environments (Richardson and McEwan, 2018). The scientific community has shown a growing interest in the relationship between leisure activities and nature-relatedness, especially in promoting physical and psychological well-being. For instance, studies have found that physical activity, especially when conducted with parents during primary school age, is positively associated with nature-relatedness (Puhakka et al., 2018), and experiencing nature is a significant motive for physical activity among adults, with green exercisers prioritizing motives related to convenience and experiencing nature over those related to physical health and sociability (Calogiuri and Elliott, 2017). Additionally, the natural environment, particularly when integrated into residential and workspaces, enhances job engagement and creativity through increased time spent outdoors and enjoyment of outdoor activities (Brossoit et al., 2024), highlighting the importance of leisure activities in natural settings in fostering a deeper connection with nature, which in turn can contribute to improved well-being and environmental attitudes.

### Flow experience

The concept known as flow experience or optimal experience refers to a state of complete immersion in a physical or cognitive action, introduced in 1975 by Csikszentmihalyi based on observations in play activities (Csikszentmihalyi, 1988). Characterized by intense concentration, enjoyment, and a sense of control (Huang and Liao, 2017), the flow experience has been extensively studied across various fields such as sports, work, education, and online video games, being recognized for its highly enjoyable and intrinsically rewarding nature (Weber et al., 2009). Several factors have been identified as antecedents of the flow experience, including difficulty-ability balance, clear goals, immediate feedback, a sense of control, and the presence of clear and unambiguous rules (Lin and Joe, 2012). Difficulty-ability balance, which refers to the match between the task’s difficulty and the individual’s skill level, suggests that flow is more likely when the challenge is high but manageable and the individual possesses the necessary skills to meet the challenge (Swann et al., 2012). Flow experience has been shown to have beneficial effects in various contexts, including work, where it is associated with increased job satisfaction, creativity, and productivity (Fullagar and Kelloway, 2009; Swann et al., 2012); sports, where it leads to increased performance and enjoyment (Swann et al., 2012); education, where it fosters engagement, motivation, and positive learning outcomes; and leisure activities, where it enhances the sense of enjoyment and satisfaction level (Huang and Liao, 2017). Furthermore, flow experiences may foster a profound connection with natural environments and motivate environmentally responsible behaviors. For instance, it was highlighted that mountain hikers experiencing flow, facilitated by their recreation specialization and perception of restorative environments, develop stronger connections to nature (Wöran and Arnberger, 2012). Similarly, it was found that environmental interpretation enhancing ecological flow experiences significantly influences visitors’ environmental attitudes and behaviors, suggesting that fostering flow experiences in natural settings can lead to increased environmental awareness and responsible actions (Tang et al., 2022).

### Environmental behavior

Environmental behavior encompasses a wide range of individual actions and decisions that directly or indirectly impact the natural environment, extending beyond mere activities to include the decision-making processes leading to actions such as waste management, recycling, energy conservation, sustainable consumption, and participation in environmental initiatives (Barr, 2007). This concept is operationalized through a multidimensional approach that considers not only observable actions but also the motivations, attitudes, and societal norms influencing these behaviors (Zhao, 2023). Understanding the complex factors driving environmental behavior is crucial for promoting sustainable practices and tackling the environmental challenges we face. Environmental behavior has been explored in depth through studies on individuals’ attachment to nature and the assessment of this attachment using psychological measures. For instance, the effects of environmental behavior on human health and well-being have demonstrated the importance of connectedness and relatedness to nature and the environment (Keaulana et al., 2021). A strong correlation was found between nature connection and environmental behavior, and this relationship has been supported by both correlational and empirical evidence (Mackay and Schmitt, 2019). It was stated that nature-relatedness refers to the cognitive, emotional, and experiential connection of individuals with the natural environment and that this connection may have an impact on environmental behavior in natural settings (Nisbet et al., 2009; Lee and Jan, 2015). Finally, it was supported that nature-relatedness and environmental concern are important factors affecting individuals’ environmental behaviors (Dornhoff et al., 2019).

### Environmental sensitivity

Environmental sensitivity encapsulates an individual’s ability to notice, process, and evaluate environmental information, fostering a deep awareness and concern for the environment that transcends mere knowledge acquisition. It involves forming a profound connection with the environment, leading to proactive preservation efforts. This concept highlights the cognitive and emotional bases motivating environmental behaviors, offering a nuanced perspective on individual engagement with environmental issues (Federal Interagency Stream Restoration Working Group, 1998). It influences attitudes toward the environment, affects health outcomes, and shapes responses to environmental stimuli, establishing itself as a crucial element of environmental stewardship (Schultz et al., 2004). Research has linked environmental sensitivity to psychological constructs like anxiety sensitivity and educational outcomes, such as active and democratic citizenship, suggesting its development is influenced by genetic and environmental factors (Ersoy, 2014). Recognizing its significance has profound implications in education, public health, and environmental management, advocating for the integration of environmental themes into educational curricula to enhance awareness and conservation behaviors (Hyde and Karney, 2001), informing public health policies aimed at mitigating pollution’s impact on mental well-being (Ventriglio et al., 2020), and tailoring strategies to engage individuals effectively in sustainable practices by considering individual differences in environmental sensitivity (Asah-Kissiedu et al., 2020).

### Communication with nature

Communication with nature refers to the various ways in which individuals interact, connect, and engage with the natural world. This interaction encompasses a broad range of experiences and practices, from passive appreciation to active participation in environmental conservation efforts. This concept signifies a deep relationship between humans and nature, characterized by mutual influence and interconnectedness (Herzog and Strevey, 2008). Studies have highlighted its significance, showing that communication with nature not only enhances personal well-being but also fosters a greater inclination toward environmental actions (Asah-Kissiedu et al., 2020). Nature-relatedness in childhood, plays a crucial role, with research indicating that children’s communication to nature is stronger when their parents value outdoor nature experiences (Chawla, 2020). This underscores the importance of early experiences and parental support in nurturing a bond with the natural world. Furthermore, the well-being benefits of communication with nature, include improved emotional functioning, life satisfaction, psychological resilience, and stress management, highlighting the potential of nature-based interventions to bolster well-being and mental health outcomes (Capaldi et al., 2015).

### Hypotheses and theoretical model

#### Nature-relatedness and flow experience

Based on the current literature, it can be hypothesized that there is a positive relationship between nature engagement and flow experience. Some studies have provided evidence to support this hypothesis. For instance, a significant positive correlation was found between nature-relatedness and vitality (Lawton et al., 2017), suggesting that people who feel connected to nature have higher feelings of energy and vitality, which is consistent with flow experiences (Peifer, 2012; Chang, 2020). Additionally, there was a significant relationship between nature-relatedness and general anxiety, immediate cognitive anxiety, and trait cognitive anxiety (Martyn and Brymer, 2016). Namely, a deeper sense of nature-relatedness is associated with a reduction in levels of general anxiety, immediate cognitive anxiety, and trait cognitive anxiety. Moreover, nature-based activities were reported to have a positive effect on reducing situational anxiety, suggesting that exposure to nature may promote a more relaxed and focused state of mind and provide a conducive environment for the flow experience (Xie et al., 2022). The current literature supports the hypothesis that attachment to nature positively affects the flow experience. Individuals who feel more connected to nature are more likely to be fully focused on their activities, lose themselves in the moment, and experience enjoyment. Therefore, the following hypothesis was formulated:

*H_*1*_*: Nature-relatedness has a positive effect on the flow experience.

#### Nature relatedness, environmental behavior, environmental sensitivity, and communication with nature

Based on the existing literature, it can be hypothesized that there is a positive relationship between nature-relatedness with environmental behavior, environmental sensitivity, and communication with nature. For example, it was found that individuals with higher nature-relatedness are more tend to environmentally friendly behaviors (Kim et al., 2020). It was suggested that commitment to nature may be related to environmental sensitivity and environmental responsibility (Zelenski and Nisbet, 2014). A partial mediation of nature-relatedness was found in the relationship between nature experiences and ecological behavior (Göker, 2022). It was also reported that nature-relatedness is associated with environmentally friendly behavior (Geng et al., 2015). Furthermore, it was investigated the effects of spending time in nature on intrinsic desires and generosity and found that exposure to nature increases individuals’ intrinsic desires (Weinstein et al., 2009). This suggests that individuals who report higher nature-relatedness may have more motivation to communicate with nature and develop a deeper relationship with their natural environment. Furthermore, it was stated that individuals who feel high levels of nature-relatedness may be more inclined to communicate with nature and recognize the connection between themselves and the natural world (Nisbet and Zelenski, 2013). This also suggests that nature-relatedness may provide a sense of calmness and relaxation, and this may facilitate communication with nature. In this context, it can be concluded that nature-relatedness positively affects environmental behavior, environmental sensitivity, and communication with nature. Therefore, the following hypotheses are as follows:

*H_*2*_*: Nature-relatedness has a positive effect on environmental behavior.

*H_*3*_*: Nature-relatedness has a positive effect on environmental sensitivity.

*H_*4*_*: Nature-relatedness has a positive effect on communication with nature.

### Mediating role of flow experience

Based on the existing literature, it can be hypothesized that flow experience has a mediating effect on the relationship between nature-relatedness and dependent variables in the study. For instance, it was found that environmental interpretation may increase the likelihood of experiencing ecological flow, which may indirectly affect individuals’ environmental attitudes and behavioral tendencies (Tang et al., 2022). It was shown that feeling connected to nature is beneficial for well-being and can lead to pro-environmental behavior (Lumber et al., 2017). In this process, the flow experience can play an important role, enabling individuals to experience an experience beyond time and space, where they lose themselves completely in their interaction with nature. This connection can encourage more environmentally responsible behavior and help individuals become more motivated to protect nature. Furthermore, it was observed that individuals who experienced high levels of flow experience during outdoor activities also had high levels of nature-relatedness (Capaldi et al., 2014). The experience of high levels of flow during outdoor activities strengthens individuals’ connection to nature, which in turn can increase their desire to value and protect it more. It was also suggested that interaction with nature can trigger the flow experience by directing physical or cognitive actions and providing interesting stimuli (Weinstein et al., 2009). In a state of flow, individuals are fully integrated in their activity, and this can increase their connectedness to nature, while encouraging more conscious and responsible behavior towards the environment. Accordingly, the current literature supports the hypothesis that flow experience plays a mediating role in the relationships between nature-relatedness and environmental behavior, environmental sensitivity, and communication with nature. Therefore, the following hypotheses are as follows:

*H_*5*_*: Flow experience has a mediating role in the relationship between nature-relatedness and environmental behavior.

*H_*6*_*: Flow experience has a mediating role in the relationship between nature-relatedness and environmental sensitivity.

*H_*7*_*: Flow experience has a mediating role in the relationship between nature-relatedness and communication with nature.

**Figure 1:**
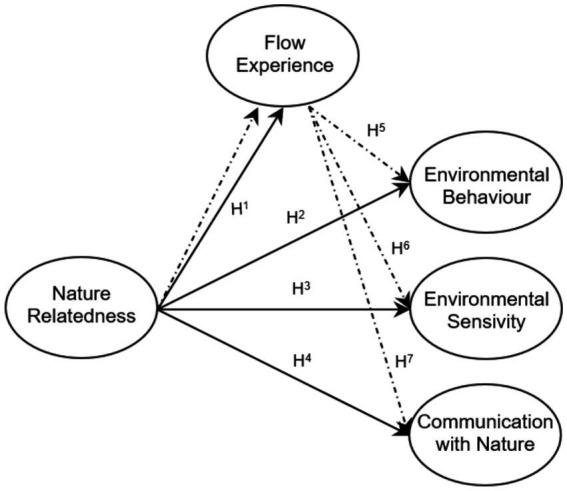
Theoretical model.

In this study, a theoretical model was created by using the theories of nature relatedness, flow experience, environmental behavior, environmental sensitivity, and communication with nature based on literature review, logical thinking, and hypotheses. The theoretical model is shown in Figure 1.

## Methods

Following the aim of the study, nature-based activities were categorized into five groups: cycling, fishing, canoeing/surfing/diving, hiking, and climbing. These activities represent a broad spectrum of nature-based leisure activities appealing to diverse demographics, including varying ages, genders, educational levels, socioeconomic statuses, and geographical regions. To gather a comprehensive sample, recruitment focused on both physical and virtual locations frequented by enthusiasts of these activities. Specifically, Turkish cycling communities on the Instagram platform were approached for cycling; anglers were contacted at the Karadeniz Ereğli district of Zonguldak province for fishing; enthusiasts of hiking and climbing were recruited from Konya province; and participants for canoeing/surfing/diving were sourced from the Foça district of Izmir province. The recruitment strategy was designed to mitigate research limitations such as time and financial constraints. Data were collected using a structured questionnaire that was distributed in these areas, ensuring a wide and representative sample of regular participants in these activities. Research protocols adhered to ethical guidelines, emphasizing voluntary participation and informed consent, while ensuring data anonymity and confidentiality.

### Questionnaire design

The questionnaire used in the research consisted of two parts. The first part included a personal information form with questions about gender, age, and regular nature-based leisure activities. The second part consisted of validated scales to measure various psychological and environmental dimensions: the ‘Nature-relatedness Scale (NRS-6)’ with 6 statements for assessing nature relatedness, the ‘Recreational Flow Experience Scale (RFES)’ with 18 items for evaluating flow experience, and the ‘Attitude Towards Ecorecreation Scale (ATES)’ with 31 statements covering environmental behavior, environmental sensitivity, and communication with nature. All scales employed a 5-point Likert-type format, ensuring consistency in response measurement across the different constructs assessed. To measure the nature relatedness, the NRS-6, which consisted of 21 statements in the original and 6 statements in the short form, was used (Nisbet and Zelenski, 2013). In the study conducted by Nisbet and Zelenski, the 6 statements and one-factor model explained 62.48% of the total variance. Some of the scale items are: “My ideal vacation spot would be in mote, wilderness area. I always think about how my actions affect the environment. My connection to nature and the environment is a part of my spirituality.” The Cronbach’s alpha internal consistency coefficient of the scale was found to be 0.96. Test–retest reliability coefficients were found between 0.80 and 0.84 for 1-month periods. The Turkish validity and reliability of NRS-6 was conducted (Şahin et al., 2015). According to the exploratory factor analysis results, a single-factor structure of 6 statements was supported, and in the confirmatory factor analysis results of the unidimensional model, the fit index values were Normed Chi-square (*χ*^2^*/df*) = 1.97, Root Mean Square Error of Approximations (RMSEA) = 0.035, Goodness-of-fit Index (GFI) = 0.96, Adjusted Goodness-of-fit Index (AGFI) = 0.96, Comparative Fit Index (CFI) = 0.97, The Normed Fit Index (NFI) = 0.96 and Standardized Root Mean Square (SRMR) = 0.04. The factor loadings of the scale ranged between 0.42 and 0.74. Cronbach’s alpha internal consistency coefficient was calculated as 0.89. The test–retest reliability coefficient was found to be 0.89. The total score that can be obtained from NRS-6 was 30.

To measure the flow experience, the RFES was used (Ayhan et al., 2020). The RFES was originally developed in Turkish. During the development phase of the RFES, an expression pool consisting of 18 items was created. According to the expert opinions, 6 statements that were not suitable for the structure of the scale were removed from the scale. Some of the scale items are as follows: “I feel like I had a positive experience during the leisure activity I participated in. I lost track of time during the leisure activity I participated in. I feel extremely motivated during the leisure activity I participated in.” At the last point, according to the results of exploratory factor analysis, the one-factor model with 9 statements explained 53.98% of the total variance. A new study was conducted to confirm the validity of the scale. According to the confirmatory factor analysis results obtained in this study, the model was defined as valid and reliable. The total score that can be obtained from the RFES was 45.

The ATES was used to measure the variables of environmental behavior, environmental sensitivity, and communication with nature (Durhan and Karaküçük, 2020). The ATES consists of a total of 6 dimensions (individual, social, environmental behavior, antipathy, environmental sensitivity, and communication with nature) and 31 statements. Some of the scale items are as follows: “Eco-recreation increases my harmony with the environment. I share my experiences regarding eco-recreation with my circle. I believe that eco-recreation is a rehabilitation tool.” In this study, environmental behavior (5 statements), environmental sensitivity (4 statements), and communication with nature (3 statements) sub-dimensions were used. The findings of the confirmatory factor analysis conducted to determine the validity of the ATES revealed that the model provided a good fit (*χ*^2^*/df* = 2.416, RMSEA = 0.061; SRMR = 0.56; NFI = 0.95, CFI = 0.97). Cronbach’s alpha internal consistency coefficient was 0.78 in the environmental behavior sub-dimension, 0.77 in the environmental sensitivity sub-dimension, and 0.70 in the communication with nature sub-dimension. The total scores that can be obtained from environmental behavior, environmental sensitivity, and communication with nature variables are 25, 20, and 15, respectively.

### Data collection process

The data collection process consisted of five parts. In the first part, Turkish cycling communities on the Instagram platform were reached via message to reach individuals engaged in cycling activities. The presidents of these communities were informed about the study, and then they were asked to share the prepared questionnaire form with the WhatsApp groups of their communities. Data were collected between 1 and 7 November 2022. In the second part, in order to reach individuals engaged in fishing activities, data were collected on the coastline of the Karadeniz Ereğli district of Zonguldak province of Turkey between 1 and 15 December 2022. In the third section, to reach individuals engaged in canoeing/surfing/diving, data were collected in Mersinaki Bay in Foça district of Izmir province, Turkey between 1–15 June 2023. In the fourth and fifth sections, the Selçuklu Mountaineering Search and Rescue Club, which serves in the central districts of Konya province of Turkey, was reached to reach individuals engaged in trekking and climbing. The club president was informed about the study, and then they were asked to share the prepared questionnaire form with the WhatsApp groups of their communities. The data were collected between 1 and 7 November 2022.

### Participants

Descriptive results for the participants were presented in Table 1. The sample of the study consisted of individuals who regularly engage in nature-based leisure activities in Turkey. The ideal sample size that can represent the research was determined using N > 50 + 8 m formula (Tabachnick et al., 2013). Since there were 27 statements in total expressing five variables in the study, according to this formula, the sample size was determined as 266 at a 95% confidence interval. However, the sample was not limited, and 379 individuals were reached in total.

**Table 1:** Descriptive results of the research group.

	*f*	%
Gender		
Male	212	55.9
Female	167	44.1
Age		
18–24	59	15.6
25–34	50	13.2
35–44	68	17.9
45–54	100	26.4
55 and older	102	26.9
Regularly participated nature-based leisure activity		
Cycling	75	19.8
Fishing	88	23.2
Canoeing, surfing and diving	53	14.0
Hiking	122	32.2
Climbing	41	10.8
Total	379	100.0

### Statistical analysis

In this study, several statistical analyses were performed to ensure the robustness and validity of the data collected. Firstly, Construct Validity was assessed using Confirmatory Factor Analysis (CFA), which confirmed the unidimensional structure of the scales used in the study. Secondly, Reliability was examined through Cronbach’s Alpha and Composite Reliability scores for each scale to ensure internal consistency. Lastly, Multiple Regression Analysis was used to explore the relationships between the variables.

## Results

### Construct validity and reliability analysis

According to the results of CFA (Table 2), the factor loadings of all measurement tools used in the study were above the acceptable level. Cronbach’s alpha results were positive (*α* ≥ 0.70). Although the measurement tools produced satisfactory results in terms of CR (CR ≥ 0.70), the AVE value for only environmental sensitivity was slightly below the recommended threshold (AVE ≥ 0.50).

**Table 2:** Construct validity and reliability results.

Variables	Phrase codes	Total	*SD*	*λ*	*α*	CR	AVE
Flow experience	FE1	41.63	0.451	0.579	0.870	0.896	0.496
	FE2			0.591			
	FE3			0.665			
	FE4			0.547			
	FE5			0.582			
	FE6			0.697			
	FE7			0.686			
	FE8			0.764			
	FE9			0.729			
Nature relatedness	NR1	27.82	0.497	0.761	0.891	0.896	0.592
	NR2			0.866			
	NR3			0.732			
	NR4			0.837			
	NR5			0.748			
	NR6			0.655			
Environmental behavior	EB1	18.36	0.925	0.761	0.885	0.894	0.629
	EB2			0.819			
	EB3			0.834			
	EB4			0.722			
	EB5			0.725			
Environmental sensitivity	ES1	17.30	0.671	0.523	0.711	0.727	0.405
	ES2			0.707			
	ES3			0.627			
	ES4			0.637			
Communication with nature	CN1	13.35	0.674	0.737	0.821	0.837	0.634
	CN2			0.898			
	CN3			0.745			

*χ*^2^	*df*	*χ*^2^*/df*	RMR	RMSEA	GFI	AGFI	CFI	NFI
576.830	307	1.879	0.036	0.048	0.901	0.879	0.948	0.896

Goodness-of-fit indexes	Perfect fit criteria	Acceptable fit criteria
*χ*^2^*/df* (Bentler, 1980; Baumgartner and Homburg, 1996)	0 ≤ *χ*^2^*/df* ≤ 2	2 ≤ *χ*^2^*/df* ≤ 3
RMR (Bentler, 1980; Baumgartner and Homburg, 1996)	0.00 ≤ RMR ≤ 0.05	0.05 ≤ RMR ≤ 0.10
RMSEA (Bentler, 1980; Baumgartner and Homburg, 1996)	0.00 ≤ RMSEA ≤0.05	0.05 ≤ RMSEA ≤0.08
GFI (Bentler, 1980; Baumgartner and Homburg, 1996)	0.95 ≤ GFI ≤ 1.00	0.90 ≤ GFI ≤ 0.95
AGFI (Bentler, 1980; Baumgartner and Homburg, 1996)	0.90 ≤ AGFI ≤1.00	0.85 ≤ AGFI ≤0.90
CFI (Bentler, 1980; Baumgartner and Homburg, 1996)	0.95 ≤ CFI ≤ 1.00	0.90 ≤ CFI ≤ 0.95
NFI (Bentler, 1980; Baumgartner and Homburg, 1996)	0.95 ≤ NFI ≤ 1.00	0.90 ≤ NFI ≤ 0.95

### Multiple regression analysis

The results of the multiple regression analysis were presented in Table 3. According to the findings, nature-relatedness was positively correlated to flow experience, environmental behavior, environmental sensitivity, and communication with nature (*p* < 0.01). Nature-relatedness explained 50.5% of flow experience, 10.8% of environmental behavior, 13.7% of environmental sensitivity, and 20.0% of communication with nature.

**Table 3:** Regression results.

Independent variable	Dependent variable	*β*	*SE*	*t*	*p*	95% C.I.	
						LLCI	ULCI
Nature relatedness	Flow experience (*R*^2^ = 0.505)	0.637	0.032	19.639	< 0.001	0.573	0.701
Nature relatedness	Environmental behavior (*R*^2^ = 0.108)	0.604	0.089	6.759	< 0.001	0.428	0.780
Nature relatedness	Environmental sensitivity (*R*^2^ = 0.137)	0.494	0.063	7.742	< 0.001	0.368	0.619
Nature relatedness	Communication with nature (*R*^2^ = 0.200)	0.599	0.061	9.714	< 0.001	0.477	0.720

Table 4 presented the total effect, direct effect, and indirect effect values between the latent variables in the structural equation model. According to the findings, nature-relatedness predicted environmental sensitivity, environmental behavior, and communication with nature and did so both directly and indirectly through the mediation of flow experience.

**Table 4:** Results regarding the mediating effect of flow experience.

Pathway	Total impact	Direct effect	Indirect effect	95% C.I.	
				LLCI	ULCI
Nature Relatedness Environmental Behavior	0.604	0.010	0.593	0.422	0.789
Nature Relatedness Environmental Sensitivity	0.494	0.209	0.285	0.141	0.401
Nature Relatedness Communication with Nature	0.599	0.289	0.309	0.139	0.448

## Discussion

In the CFA process, the lower limit of the factor loadings was determined using Norman & Streiner’s formula x = (5.152/√N−2) (Norman and Streiner, 2008). According to this formula, it was suggested that factors with a value higher than 0.26 are valid. The factor loadings of the statements reflecting all variables used in the research comply with this proposition. The fit index values of the model created for the aims of the research met the suitability criteria (Bentler, 1980; Baumgartner and Homburg, 1996). In the reliability process, on the other hand, the Cronbach’s alpha internal consistency values of all the instruments used in the study were above the recommended value of 0.70 (Hair et al., 2019). Furthermore, except for environmental sensitivity, all measurement tools used in the study were reliable in terms of CR and AVE values. The AVE value of environmental sensitivity (0.40) was lower than recommended (Hair et al., 2019). However, it has been suggested to make an evaluation by considering factors such as factor analysis and Cronbach’s Alpha (Churchill, 1979; Fornell and Larcker, 1981; Hair, 2009; Hair et al., 2011). Therefore, environmental sensitivity can be considered reliable.

Following multiple regression analysis, the hypothesized positive effects of nature-relatedness on psychological and environmental outcomes were supported. Specifically, we confirmed that nature-relatedness positively influences the flow experience (H_1_), enhances environmental behavior (H_2_), increases environmental sensitivity (H_3_), and improves communication with nature (H_4_). Additionally, our findings affirmed the mediating role of the flow experience in strengthening the relationship between nature-relatedness and key environmental behaviors (H_5_), sensitivity (H_6_), and communication (H_7_).

Our research provides compelling evidence that nature-relatedness significantly augments the flow experience, enabling individuals to engage more profoundly with their natural surroundings. This engagement is not merely a passive occurrence but an active emotional and motivational enhancement that heightens the overall experience. Positive emotions such as joy, excitement, and pleasure, which are frequently encountered during interactions with nature, play a pivotal role in this process. These emotions not only elevate motivational tendencies but also substantially encourage the onset and continuation of flow states during nature-based activities, offering a richer and more satisfying experience (Sheldon, 2011). Additionally, the intrinsic interest and longing for nature that characterize individuals with high nature-relatedness suggest a more substantial likelihood of seeking and participating in activities that induce flow in natural settings, thereby creating a self-reinforcing cycle of engagement and enjoyment (Nisbet and Zelenski, 2013; Martyn and Brymer, 2016; Dean et al., 2018).

Our findings elucidate the positive impact of nature-relatedness on environmental behaviors, indicating that individuals deeply connected to nature are more likely to exhibit environmental sensitivity and proactive behaviors toward nature conservation. This relationship is mediated through the enhanced mood and general well-being that nature-relatedness fosters, which, in turn, encourages a more active and positive interaction with the environment. Such behaviors include efforts to minimize adverse impacts on nature and increased participation in activities that promote environmental sustainability (Zelenski and Nisbet, 2014; Kim et al., 2020; Whitburn et al., 2020). The enhancement of positive emotions through increased nature-relatedness not only improves the mood of individuals but also propels them toward a more robust engagement with environmental protection efforts, thereby contributing to overall environmental health and sustainability (Nisbet et al., 2019).

The role of flow experience as a mediator in the relationship between nature-relatedness and environmental behavior is particularly noteworthy. Flow experiences in natural settings appear to be a critical motivating factor for environmentally friendly behaviors, acting as a positive psychological mechanism that enhances an individual’s propensity to engage in and promote sustainable practices. This mediation suggests that the immersion and enjoyment derived from nature can significantly influence environmental attitudes and actions, leading to more responsible behaviors and a greater willingness to advocate for and participate in environmental conservation (Byrka et al., 2010; DeVille et al., 2021; Pasca, 2022). The emotional dynamics involved, such as the pride felt from engaging in environmentally positive behaviors and the reduction of environmentally harmful actions, highlight the complex interplay between psychological states and environmental ethics.

This study substantially contributes to the existing literature on nature-based leisure by delineating how deep emotional and psychological connections with nature can amplify the benefits derived from natural environments. For practitioners and providers of nature-based leisure, these insights emphasize the importance of designing experiences that not only engage individuals but also foster a lasting relationship with nature. By enhancing nature-relatedness and facilitating flow experiences, we can encourage more sustainable environmental behaviors and greater well-being among participants. This approach can provide practical benefits for both individuals and communities, promoting a sustainable and enriching engagement with the natural world.

**Table 5:** Hypothesis results.

Hypothesis	Results
H_1_: Nature-relatedness has a positive effect on the flow experience	Supported
H_2_: Nature-relatedness has a positive effect on environmental behavior	Supported
H_3_: Nature-relatedness has a positive effect on environmental sensitivity	Supported
H_4_: Nature-relatedness has a positive effect on communication with nature	Supported
H_5_: Flow experience has a mediating role in the relationship between nature-relatedness and environmental behavior	Supported
H_6_:Flow experience has a mediating role in the relationship between nature-relatedness and environmental sensitivity	Supported
H_7_: Flow experience has a mediating role in the relationship between nature-relatedness and communication with nature	Supported

## Limitations

This study leverages a robust methodological framework to explore the impact of nature-based activities on environmental behaviors. By categorizing nature-based activities into diverse groups such as cycling, fishing, canoeing, surfing, diving, hiking, and climbing, the research captures a broad spectrum of interactions with nature, appealing to a wide demographic cross-section. The recruitment strategy was meticulously designed to ensure a comprehensive and representative sample by targeting both physical locations and virtual communities actively engaged in these activities across different regions of Turkey. Such a strategy not only enhances the diversity of the sample but also bolsters the generalizability of our findings across various socio-demographic groups. Additionally, the structured questionnaire facilitated detailed and consistent data collection, while adherence to ethical guidelines ensured the integrity and confidentiality of the participant responses, thereby strengthening the validity and reliability of our study results.

Notwithstanding the strengths of the study, there are some limitations. The limitations of this study primarily relate to the demographic characteristics and geographical locations of the participants. The majority of the participants are from a specific geographical area, which may limit the generalizability of the results. Additionally, the fact that participants regularly engage in nature-based leisure activities may make it difficult to generalize the findings to the entire population. Secondly, the study was conducted using structural equation modeling, a method that relies on assumptions made on relational data. Therefore, the findings of the study provide insights into the relationships among variables rather than directly proving causality. Thirdly, the subjective nature of psychological measures such as nature relatedness, flow experience, and environmental behaviors could introduce bias in participants’ responses. Lastly, since the research focuses solely on individuals participating in nature-based leisure activities, the experiences of those who are not interested in such activities have not been considered.

## Conclusion

### Theoretical implications

This study enriches our understanding of how nature-relatedness can significantly influence environmental behaviors, illustrating its importance as a theoretical construct in environmental psychology. By establishing a link between nature-relatedness and flow experiences, the research underlines the role of positive emotions in fostering deeper engagement with the natural world. These findings suggest that emotional and motivational enhancements experienced during interactions with nature are crucial for promoting sustained environmental behaviors. Furthermore, the evidence that flow experience acts as a mediator in this relationship provides a novel perspective on how psychological states can influence environmental ethics and actions. This highlights the potential for interventions aimed at increasing nature-relatedness to not only improve individual well-being but also to encourage broader environmental conservation efforts. The study’s insights can guide future research in exploring the mechanisms through which nature-based interventions can be optimized to foster both personal and ecological benefits.

### Practical implications

This study underscores the benefits of nature-relatedness for enhancing flow experience and environmental behaviors, suggesting actionable strategies for organizations in both the public and private sectors. Managers can create positive impacts by constructing nature-based recreation areas, promoting nature-related activities, and ensuring easy access to green spaces. Additionally, integrating training on environmentally friendly practices and supporting sustainability initiatives can further foster environmental stewardship. In workplace settings, providing access to natural spaces can significantly reduce work-related stress and enhance employee well-being, thereby boosting productivity. Health organizations and community managers can also leverage these findings by facilitating public access to natural areas, thus contributing to public health through stress reduction and mental relaxation. For the tourism and recreation sectors, emphasizing and responsibly managing nature-based leisure activities can attract more visitors, enhance their experience, and simultaneously benefit environmental conservation. These approaches collectively underline the practical benefits of nurturing a deeper connection with nature to improve individual well-being and promote sustainable practices.

### Future research

Future research should explore the diverse psychological effects of various less common nature-based leisure activities, such as camping, birdwatching, or stargazing, to better understand how different experiences influence psychological outcomes. Investigating the factors that enhance the likelihood of experiencing flow during these activities—including the influence of personality traits, motivation, experience levels, and perceptions—is crucial for a more nuanced understanding. Longitudinal studies are also needed to examine changes in nature-relatedness over time and its long-term impacts on psychological well-being. Additionally, more detailed investigations into how nature-based leisure impacts stress levels, mood, depression, anxiety, and physical health parameters like heart rate and blood pressure would provide a deeper insight into the health benefits of these activities. Such research will not only expand our knowledge but also inform the development of targeted interventions to maximize the benefits of engagement with nature.

## Data availability statement

The original contributions presented in the study are included in the article/supplementary material, further inquiries can be directed to the corresponding author.

## Ethics statement

This study was approved by the Social and Human Sciences, Scientific Research Ethics Committee in Necmettin Erbakan University on 08 Sep 2023 with decision number 2023/355. The studies were conducted in accordance with the local legislation and institutional requirements. Written informed consent for participation in this study was provided by the participants.

## Author contributions

AA: Writing – review & editing, Writing – original draft, Visualization, Validation, Supervision, Software, Resources, Project administration, Methodology, Investigation, Funding acquisition, Formal analysis, Data curation, Conceptualization. MD: Writing – review & editing, Writing – original draft, Visualization, Validation, Supervision, Software, Resources, Project administration, Methodology, Investigation, Funding acquisition, Formal analysis, Data curation, Conceptualization. AY: Writing – review & editing, Writing – original draft, Visualization, Validation, Supervision, Software, Resources, Project administration, Methodology, Investigation, Funding acquisition, Formal analysis, Data curation, Conceptualization. HG: Writing – review & editing, Writing – original draft, Visualization, Validation, Supervision, Software, Resources, Project administration, Methodology, Investigation, Funding acquisition, Formal analysis, Data curation, Conceptualization. CA: Writing – review & editing, Writing – original draft, Visualization, Validation, Supervision, Software, Resources, Project administration, Methodology, Investigation, Funding acquisition, Formal analysis, Data curation, Conceptualization. HS: Writing – review & editing, Writing – original draft, Visualization, Validation, Supervision, Software, Resources, Project administration, Methodology, Investigation, Funding acquisition, Formal analysis, Data curation, Conceptualization. ÖI: Writing – review & editing, Writing – original draft, Visualization, Validation, Supervision, Software, Resources, Project administration, Methodology, Investigation, Funding acquisition, Formal analysis, Data curation, Conceptualization. DD: Writing – review & editing, Writing – original draft, Visualization, Validation, Supervision, Software, Resources, Project administration, Methodology, Investigation, Funding acquisition, Formal analysis, Data curation, Conceptualization. LS: Writing – review & editing, Writing – original draft, Visualization, Validation, Supervision, Software, Resources, Project administration, Methodology, Investigation, Funding acquisition, Formal analysis, Data curation, Conceptualization.
